# G Protein-Coupled Estrogen Receptor in Cancer and Stromal Cells: Functions and Novel Therapeutic Perspectives

**DOI:** 10.3390/cells10030672

**Published:** 2021-03-17

**Authors:** Richard A. Pepermans, Geetanjali Sharma, Eric R. Prossnitz

**Affiliations:** 1 Division of Molecular Medicine, Department of Internal Medicine, University of New Mexico Health Sciences Center, Albuquerque, NM 87131, USA; rpepermans@salud.unm.edu (R.A.P.); gsharma@salud.unm.edu (G.S.); 2 Center of Biomedical Research Excellence in Autophagy, Inflammation and Metabolism, University of New Mexico Health Sciences Center, Albuquerque, NM 87131, USA; 3 University of New Mexico Comprehensive Cancer Center, University of New Mexico Health Sciences Center, Albuquerque, NM 87131, USA

**Keywords:** estrogen, GPER, cancer, stroma, metabolism, obesity

## Abstract

Estrogen is involved in numerous physiological and pathophysiological systems. Its role in driving estrogen receptor-expressing breast cancers is well established, but it also has important roles in a number of other cancers, acting both on tumor cells directly as well as in the function of multiple cells of the tumor microenvironment, including fibroblasts, immune cells, and adipocytes, which can greatly impact carcinogenesis. One of its receptors, the G protein-coupled estrogen receptor (GPER), has gained much interest over the last decade in both health and disease. Increasing evidence shows that GPER contributes to clinically observed endocrine therapy resistance in breast cancer while also playing a complex role in a number of other cancers. Recent discoveries regarding the targeting of GPER in combination with immune checkpoint inhibition, particularly in melanoma, have led to the initiation of the first Phase I clinical trial for the GPER-selective agonist G-1. Furthermore, its functions in metabolism and corresponding pathophysiological states, such as obesity and diabetes, are becoming more evident and suggest additional therapeutic value in targeting GPER for both cancer and other diseases. Here, we highlight the roles of GPER in several cancers, as well as in metabolism and immune regulation, and discuss the therapeutic value of targeting this estrogen receptor as a potential treatment for cancer as well as contributing metabolic and inflammatory diseases and conditions.

## 1. Introduction

Estrogen, the female sex hormone that is best known for its role in female reproduction and mammary gland development, also plays a role in many other physiological systems, including metabolism and the cardiovascular and immune systems [1,2]. In addition to its role in females, estrogen also plays a role in male physiology [3]. Besides regulating tumor cell properties, it also has effects on diverse cells of the tumor microenvironment or stroma, including fibroblasts, immune cells, and adipocytes [4]. It exerts its function through three known estrogen receptors (ERs): ERα, ERβ, and the G protein-coupled estrogen receptor (GPER, formerly known as GPR30) [5,6,7]. Aside from its physiological roles, estrogen is also involved in various diseases, among the best-known being breast cancer [8]. For decades, estrogen has been recognized to be the major driver of ER-positive breast cancers through the activation of ERα [9]. This has led to FDA approval of several ERα-targeting drugs, such as tamoxifen and fulvestrant, and the development of various next-generation ERα-targeting antagonists in various stages of clinical development [10,11,12,13,14,15,16].

Although ERα is the main driver of the majority (~70%) of primary breast cancers, GPER, over the past decade, has gained interest as a potential contributor to the development of endocrine resistance in the breast cancer relapse setting [9,17,18]. This is in part due to observations that tamoxifen, a standard-of-care drug for ERα-positive breast cancers, cross-activates GPER [19,20]. GPER has also been implicated in other cancers as either a pro- or anti-tumorigenic factor and observations, specifically in the melanoma field, have led to the initiation of a Phase I clinical trial for a GPER-targeted drug (NCT04130516) [21,22,23].

In addition to cancer, growing evidence reveals roles for GPER in normal and pathophysiological metabolism [24,25,26,27,28]. Obesity has reached epidemic proportions and increases the risk of conditions associated with metabolic syndrome, namely high blood sugar (i.e., hyperglycemia, in the form of diabetes), high blood pressure (hypertension), and abnormal lipid levels (dyslipidemia). Furthermore, obesity also increases the risk of developing many types of cancer, including pancreatic and liver cancer [29]. Importantly, GPER has been shown to regulate body weight and glucose/lipid homeostasis and its activation exerts anti-obesity and anti-diabetic effects in multiple murine models, making it a potential therapeutic target for treating obesity and/or diabetes [25].

In this review, we discuss the direct roles of GPER in several cancers, as well as indirect roles based on obesity and metabolism- and immune-related stromal cells, suggesting multiple therapeutic roles for GPER-targeted drugs in the treatment of cancer.

## 2. Estrogen Receptors

Estrogen has three known receptors in the body: ERα, ERβ, and GPER. ERα and ERβ, the so-called classical ERs, are nuclear hormone receptors that act largely as ligand-activated transcription factors [30,31,32]. They are expressed in various tissues with some tissues expressing both receptor subtypes, while others express predominantly one subtype. For example, ERα is expressed in the breast (luminal epithelial cells), bone, and uterus whereas ERβ is expressed in the breast (stromal myoepithelial cells), colon, and lung [32,33,34]. ERα and ERβ regulate gene expression through either binding directly to DNA or indirectly through recruitment to other transcription factors [1,32]. This gene regulation results in either an increase or decrease in target gene expression, with the majority of target genes in fact showing a decrease in gene expression [35]. Aside from their ability to regulate genomic signaling (on the scale of tens of minutes to hours), ERα and ERβ also induce rapid, non-genomic signaling (on the scale of seconds to minutes), leading to multiple cellular responses, including activation of kinase pathways and Ca^2+^ mobilization [36,37,38,39]. This rapid signaling is believed to occur through a fraction of splice variants of ERα and ERβ or palmitoylated receptors at the plasma membrane rather than through the full-length cytoplasmic/nuclear versions of the receptors that are chiefly responsible for the genomic signaling [37,38].

Unlike the classical ERs, the third known estrogen receptor, GPER, is a G protein-coupled receptor (GPCR) that predominantly induces rapid, non-genomic estrogen signaling [7]. Originally cloned in the late 1990s, its activation leads to a multitude of downstream, rapid signaling events, including Ca^2+^ mobilization, cyclic adenosine monophosphate (cAMP) synthesis, and activation of kinase pathways, such as the phosphatidylinositol-3-kinase (PI3K) and mitogen-activated protein kinase (MAPK) pathways [7,40,41,42,43]. Interestingly, GPER appears not to directly activate the PI3K and MAPK signaling pathways, but rather leads to their activation via the trans-activation of the epidermal growth factor receptor (EGFR) in a mechanism involving the cleavage of heparin-bound EGF on the plasma membrane [41]. GPER is not unique in this mechanism of cell activation as many GPCRs trans-activate EGFR [44,45,46]. This was an interesting discovery for GPER given that, in many cell types, GPER is mainly localized to the endoplasmic reticulum and not to the plasma membrane, where EGFR is found [7]. However, several groups have reported lower levels of GPER at the plasma membrane in addition to intracellular compartments (endoplasmic reticulum and Golgi apparatus), even including the nucleus [47,48,49,50]. In the nucleus, it has been hypothesized to act as a transcription factor, possibly in a complex with EGFR, but the mechanism by which this could occur is not understood [50]. Although it remains unclear whether GPER can act as a transcription factor, GPER can regulate gene expression, through signaling pathways involving activation of transcription factors such as cAMP-response element binding protein (CREB); however, gene expression changes in response to GPER generally involve far fewer genes than those of ERα and ERβ [40]. GPER, through its activation by estrogen, induces the expression of genes, such as c-*fos*, in various cell types, including thyroid [51], endometrial [52], and ERα/β-negative/GPER-positive breast cancer cell lines [53]. Several of these reports have also shown that knockdown of GPER attenuates E2-induced expression of c-*fos*. Aside from c-*fos*, activation of GPER also induces expression of genes such as *CCN2* (connective tissue growth factor (CTGF)) and *Bcl-2* [40,53].

ERα, ERβ, and GPER can all induce genomic and non-genomic signaling pathways, albeit to different extents, with some of these pathways overlapping. Therefore, it can be difficult to distinguish an ER-induced signal from a GPER-induced signal. Selective compounds to these receptors have aided in better understanding the individual roles of these receptors.

## 3. Compounds That Selectively Target GPER

For decades, small molecule compounds have been used to study the roles of individual receptors within a receptor family. In the case of the classical ERs, this has been challenging due to large structural similarities between the ligand binding pockets of ERα and ERβ, resulting in a high degree of ligand cross-reactivity between the two receptor subtypes [19,32]. Nevertheless, ER subtype-biased ligands, such as propylpyrazoletriol (PPT) and diarylpropionitrile (DPN), have been developed [54,55]. PPT acts as an ERα-selective agonist that shows ~410-fold binding selectivity towards ERα over ERβ whereas DPN, an ERβ-selective agonist, exhibits ~70-fold selectivity to ERβ over ERα.

Despite the overall structural differences between the classical estrogen receptors (transcription factors) and GPER (a GPCR), cross-reactivity of ERα/β ligands towards GPER is also prevalent. In fact, until recently, ERα/β ligands, including the clinically-used ERα-targeting drugs tamoxifen, raloxifene, and fulvestrant, show binding and/or activity towards GPER [19,20]. This lack of ER ligand selectivity has been challenging when studying estrogen signaling, due to partially overlapping signaling pathways between the classical ERs and GPER. We recently identified the first known ERα/β-selective ligand, termed AB-1, that shows negligible binding to GPER at concentrations up to 10 μM [56]. It acts as an agonist of ERα and ERβ, while neither inducing nor inhibiting GPER-mediated signaling pathways. Due to the prior lack of truly ERα/β-selective compounds, such selective compounds should aid in better differentiating the roles of ERα/β versus GPER in systems that express both receptors.

Compounds with an inverse binding profile (i.e., high selectivity for GPER over ERα/β) also exist. In 2006, the first GPER-selective agonist G-1 was identified, with high binding affinity towards GPER and negligible binding to ERα and ERβ at concentrations up to 10 μM [36]. This was followed by the discovery of the first GPER-selective antagonist, G15 in 2009 [57], and the even more selective GPER antagonist, G36 in 2011 [58]. These compounds show negligible or no binding and/or activity towards the classical ERs at concentrations up to 10 μM. In 2012, Maggiolini and colleagues identified two GPER-selective agonists, termed GPER-L1 and GPER-L2, that display ~100-fold poorer affinity for GPER versus G-1 [59]. Recently, following an in silico pharmacophore screen, several indole-thiazole derivates were identified that act as selective GPER agonists [60]. These compounds show no binding to ERα/β and can induce cAMP synthesis in HL-60 cells, which can be blocked by co-treatment with G15. Whether these indole-thiazole derivates show any agonism in other GPER-mediated pathways is unknown.

## 4. GPER in Breast and Other Cancers

### 4.1. Breast Cancer

ERα-positive breast cancers make up the majority of breast cancer subtypes seen in the clinic [9]. Endocrine therapy, using selective estrogen receptor modulators (SERMs), such as tamoxifen, selective estrogen receptor downregulators (SERDs), such as fulvestrant, or aromatase inhibitors (AIs), has prolonged the lives of millions of ERα-positive breast cancer patients and has been a great clinical success [10]. However, 30% of women develop endocrine resistance to their therapy, with some reports indicating this number to be even higher [10,61]. Several mechanisms have been described to account for the development of endocrine resistance [18,62,63,64,65]. In the case of resistance to tamoxifen, a standard-of-care drug in the clinic, numerous reports, both clinical and non-clinical, have pointed to GPER as a potential player in the development of resistance. Specifically, increased GPER expression was observed in breast cancer metastases compared to their matched primary tumors [17,66]. Increased GPER expression was only observed in metastatic tumor samples of women who had been treated with tamoxifen, but not in metastases of women who were not prescribed tamoxifen, indicating a possible role for GPER in the development of relapse breast cancer [66]. To this end, GPER shows a positive association with metastasis [67], which is supported by the observation that in a breast cancer mouse model, GPER-deficiency results in decreased metastases to the lung [68]. Furthermore, GPER expression correlates negatively with progression-free survival [66], but this is contradicted by findings describing a non-statistically significant, positive correlation between GPER expression and disease-free progression (*p* = 0.07–0.12) [69]. Primary ERα-positive breast tumors, that co-express GPER, shrink significantly more when the patient is treated with an AI-based primary endocrine therapy versus a tamoxifen-based primary therapy and this difference is absent in GPER-negative primary ERα-positive breast tumors [70]. Moreover, an AI-based therapy resulted in better disease-free progression for breast cancer patients compared to a tamoxifen-based therapy [69]. The observed difference between AI and tamoxifen treatment could be due to the different mechanisms of action of the drugs, namely AIs decrease circulating E2 levels (and do not affect GPER directly), whereas tamoxifen directly inhibits ERα and directly activates GPER [64]. This activation of GPER leads to pro-survival signaling in cancer cells; possibly decreasing the overall efficacy of tamoxifen compared to an AI. Collectively, these multiple lines of clinical data suggest a role for GPER in the development or sustainability of tamoxifen resistance.

**Figure 1:**
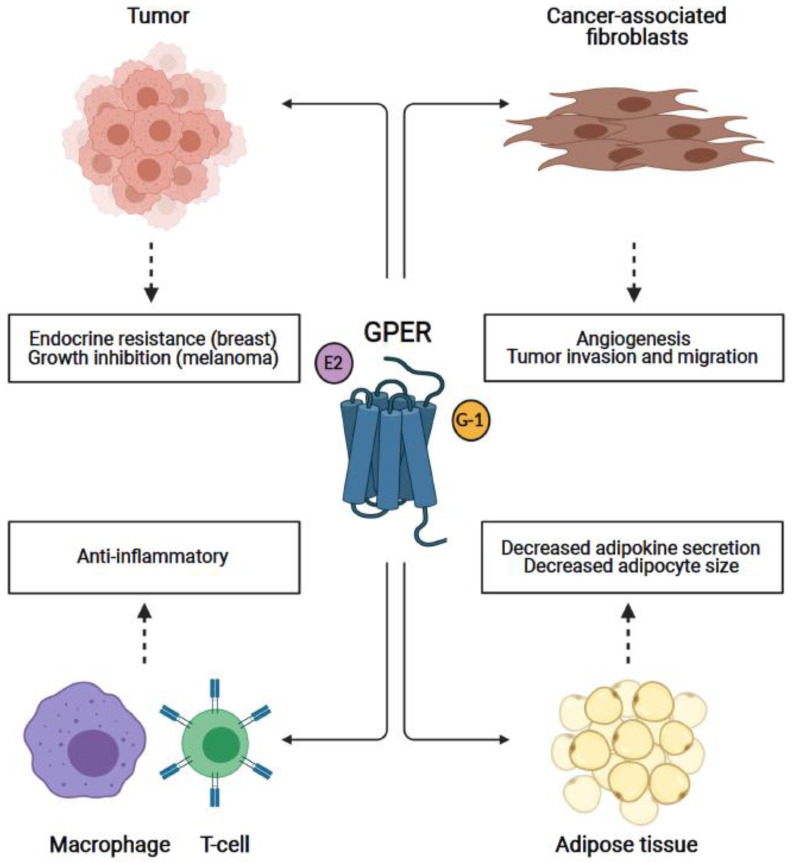
Effects of G protein coupled estrogen receptor (GPER) signaling in multiple tissue types that affect carcinogenesis. GPER signaling induces various responses in different tissue types that can promote or inhibit tumor growth and progression. In estrogen receptor (ER)-positive breast cancers, GPER activation may contribute to the development of endocrine resistance. However, in a melanoma setting, GPER signaling (through G-1 activation) inhibits tumor growth and increases the efficacy of anti-programmed cell death protein-1 (PD-1) immunotherapy. In cancer-associated fibroblasts (in breast cancer), GPER signaling leads to a pro-tumorigenic environment through increased secretion of angiogenic (e.g., vascular endothelial growth factor (VEGF)) and migratory (e.g., connective tissue growth factor (CTGF)) factors that promote tumor angiogenesis, migration, and invasion. In immune cells, GPER plays an anti-inflammatory role by decreasing the secretion of various pro-inflammatory cytokines (e.g., interleukin-1β (IL-1β), tumor necrosis factor-α (TNF-α) and interferon-γ (IFN-γ)), which could prove to be of therapeutic value in carcinogenesis and autoimmune diseases, such as multiple sclerosis, as well as in adipose tissue resulting in reduced adipokine secretion. Furthermore, G-1-induced GPER signaling leads to reduced adiposity and smaller adipocytes, which in conjunction with decreased adipokine secretion, could limit availability of adipokines and nutrients to tumors, thereby inhibiting tumor growth and progression.

*In vitro* data also support a role for GPER in the development of tamoxifen-resistance. Tamoxifen induces the *in vitro* proliferation of tamoxifen-resistant MCF-7 cells through a GPER-dependent pathway [17,18]. This proliferative effect was blocked through GPER knockdown or by co-treatment with the GPER-selective antagonist, G15 [17,57]. Tamoxifen also induces, through a GPER-dependent pathway, proliferation (or activation of proliferative factors) of other cell types, including endometrial [20,71] and thyroid [51] cancer cells. Such activation of GPER by tamoxifen is supported by binding data showing that tamoxifen binds and cross-activates GPER [19,20,72]. Aside from inducing proliferation, tamoxifen-induced cross-activation of GPER also induces breast cancer cell migration [73] and increases aromatase expression in tamoxifen-resistant cells [74]. The latter mechanism could induce an increase in local estrogen levels sufficient to counteract the inhibitory effects of tamoxifen in relapse tumors. *In vivo*, GPER also contributes to tamoxifen resistance in MCF-7 cells. Tamoxifen-resistant xenografts became sensitive to tamoxifen when mice were treated with a combination of tamoxifen and G15 [17]. Importantly, these tumors were unaffected by the individual monotherapies, thereby implicating GPER in the observed tamoxifen resistance. Collectively, the aforementioned observations implicate GPER in the development and/or sustainability of tamoxifen resistance in ERα-positive breast cancers (Figure 1).

### 4.2. Melanoma

Historically, men have had both a higher risk of developing melanoma and a worse prognosis for this disease than women [75,76]. This had led to the hypothesis that sex hormones play a role in the observed sex differences in both melanoma incidence rates as well as outcomes. A role for sex hormones in melanocyte function is suggested by their association with hyperpigmentation (melasma), in which women commonly develop darker pigmentation during pregnancy, when estrogen levels are elevated [77]. To this end, two groups have shown, using both genetic and pharmacological approaches, that GPER is required for estrogen-mediated melanogenesis (pigment formation), but whether GPER plays a role in melanoma, was unknown [78,79]. In the clinic, tamoxifen provides a benefit, in combination with chemotherapy, in the treatment of metastatic melanoma in women [80]. However, these observations have been contradicted by a meta-analysis showing that while tamoxifen does improve initial response to chemotherapy, it does not reduce the overall 1- and 2-year mortality in advanced melanoma [81]. Interestingly, the meta-analysis study did report better response rates in trials that consisted predominantly of women.

*In vitro* data support the observations that tamoxifen has some benefit in treating melanoma. For example, cell-based data reveal that tamoxifen inhibits proliferation of human and mouse melanoma cells, while also inhibiting metastasis of melanoma *in vivo* [82,83,84,85]. Furthermore, treating murine melanoma cells with the GPER-selective agonist, G-1, inhibits proliferation of these cells and treatment with tamoxifen also results in similar cellular responses [84]. Importantly, knockdown of GPER abrogated the observed effects induced by both G-1 and tamoxifen, indicating that the anti-proliferative effects of these compounds occurred through a GPER-mediated pathway, implying that activation of GPER brought about anti-proliferative effects in melanoma.

In the past decade, immune checkpoint inhibition has produced great clinical benefits for the treatment of advanced melanoma using agents such as ipilimumab [86,87]. Nevertheless, about 50% of melanoma patients do not respond to this class of treatment [88]. Ridky and colleagues examined whether estrogen, specifically through GPER, could impact the efficacy of anti-PD-1 immunotherapy in melanoma therapy [22]. Co-treatment with an anti-PD-1 antibody and G-1 not only shrank tumors, but also significantly improved survival of melanoma-bearing mice. They identified that activation of GPER by G-1, in melanoma xenografts, induces a decrease in c-Myc levels, which results in *decreased* cell surface expression of programmed cell death ligand-1 (PD-L1) and *increased* cell surface levels of human leukocyte antigen (HLA), which together lead to improved immune recognition of melanoma tumor cells. These observations suggest great promise in targeting GPER for the treatment of melanoma in combination with established anti-PD-1 immunotherapies (Figure 1). In fact, this work was has led to the initiation of the first Phase I clinical trial of G-1 (NCT04130516). That this combination therapy may extend beyond melanoma is suggested by murine models of pancreatic cancer that also revealed promising results [89].

### 4.3. Other Cancers

Aside from its expression in breast cancer and melanoma, GPER is also expressed in numerous other cancer cell lines and tumors, including endometrial [20,52,90], thyroid [51], ovarian [91,92], testicular [93,94], prostate [95], pancreatic [89], and lung [2,96]. Activation of GPER generally promotes proliferation and proliferative signaling pathways in endometrial [52], thyroid [51], and ovarian [91] cancer cell lines, although inhibition of proliferation has been reported in breast [97] and pancreatic cell lines [89]. The majority of these findings employed cell lines, so the role of GPER remains unclear in certain primary tumors types. Importantly, GPER expression in endometrial [98] and ovarian [99] tumors predicts poor survival, suggesting a pro-tumorigenic role for GPER in these types of tumors. GPER also appears to play a pro-tumorigenic/pro-survival role in certain cancer stem cells (CSCs), specifically breast CSCs [100]. Activation of GPER in ER-negative/progesterone receptor-positive breast CSCs increases the viability of these breast CSCs via a GPER/(protein kinase A (PKA)/BCL2-associated agonist of cell death (BAD) pathway [100]. Importantly, knockdown of GPER in these CSCs decreased their viability and tumorigenicity, suggesting a potential role for GPER as a potential therapeutic target for targeted killing of breast CSCs.

## 5. GPER in Cancer-Associated Fibroblasts

Fibroblasts, specifically cancer-associated fibroblasts (CAFs), make up a significant fraction of stromal cells in the tumor microenvironment and play an important role in various stages of cancer progression [101]. GPER is expressed in breast CAFs, suggesting a further role for GPER, through CAFs, in breast tumor progression [50,102,103]. Both estrogen and G-1 induce increased expression of hypoxia inducible factor-1α (HIF-1α) and the angiogenic vascular endothelial growth factor (VEGF) in breast CAFs, with the latter occurring via a HIF-1α-dependent mechanism [104]. Increased VEGF expression promotes tumor angiogenesis; thus GPER may contribute to such a pro-tumorigenic environment [105]. In a recent report, insulin growth factor 1 (IGF1) induced upregulation of GPER, HIF-1α, and VEGF in breast CAFs [106]. Importantly, knockdown of GPER or HIF-1α abolished the observed IGF1-induced upregulation of VEGF, further demonstrating the contribution of GPER in a CAF-induced angiogenic tumor microenvironment.

In addition to contributing to a pro-angiogenic microenvironment, GPER, through breast CAFs, has also been implicated in promoting tumor progression by increasing migration and invasion of breast cancer cells (Figure 1). A pro-migratory role for GPER in breast CAFs was demonstrated as a result of the hypoxia-driven expression of VEGF, interleukin-6 (IL-6), and CTGF, with enhanced invasion of breast cancer cells occurring in a GPER/CTGF-dependent manner [107]. Estrogen and G-1 both increase expression of fibroblast growth factor 2 (FGF2) in breast CAFs [108], an effect inhibited by co-treatment with G15 or knockdown of GPER. Importantly, conditioned medium from these stimulated CAFs induced activation of the FGF receptor 1 (FGFR1) on triple negative breast cancer cells *in vitro*, which subsequently promoted increased cell migration and invasion. A similar paracrine signaling mechanism involves GPER-mediated increases in expression of interleukin 1 beta (IL-1β) and IL-1 receptor 1 (IL-1R1) on CAFs and triple-negative breast cancer cells, respectively, resulting in increased migration of breast cancer cells [109]. Collectively, these reports suggest that breast CAFs, through a GPER-mediated pathway, can induce paracrine signaling that promotes tumor migration and invasion.

Breast CAFs may also promote tumor progression by increasing local concentrations of estrogen. Tamoxifen and G-1 induce increased aromatase expression and as a consequence increased estrogen production in breast CAFs, effects abolished by knockdown of GPER [102]. Similar observations by others suggest such mechanisms may contribute to clinically-observed tamoxifen resistance [74]. Additionally, increased local estrogen levels could promote the growth of primary ERα-positive breast tumors, thus driving tumor progression.

## 6. GPER in the Immune System

Stromal cells in the tumor microenvironment also include a wide variety of immune cells, which play a role not only in elimination of cancer cells, but also promoting tumor formation and progression [110,111]. For example, tumor-associated macrophages contribute to tumor progression and metastasis by secreting multiple growth factors and inflammatory cytokines into the tumor microenvironment [112,113]. Estrogens display multiple effects on the immune system, including both development and function [114,115,116]. ERα, ERβ, and GPER knockout (KO) mouse studies revealed that ERα mediates an early developmental blockage of thymocytes, whereas GPER is required for apoptosis of T cell double-positive thymocytes [117]. The latter observation could be mimicked by systemic *in vivo* treatment with the GPER-selective agonist, G-1, which induced thymic atrophy and thymocyte apoptosis in mice, while not altering the early developmental blockage of thymocytes mediated by ER. GPER deficiency also leads to a lower frequency of T cells, specifically CD62L-expressing T cells, in both male and female mice, suggesting that GPER deficiency impairs production of T cells in the thymus [118]. An anti-inflammatory role for GPER is further supported by G-1’s ability to expand regulatory T cell populations [119] and induce secretion of the anti-inflammatory cytokine, IL-10, from Th17 polarized cells [120].

Estrogens have been gaining more interest as potential anti-inflammatory therapeutic agents for autoimmune diseases, in particular multiple sclerosis (MS) [121]. In the experimental autoimmune encephalomyelitis (EAE) mouse model of MS, estrogen and G-1 provide a protective role against the disease by enhancing the suppressive activity of CD4^+^Foxp3^+^ T regulatory cells, through a GPER- and PD-1-dependent pathway [122]. G-1 provides further benefit in the EAE model by decreasing the levels of pro-inflammatory cytokines, such as IFN-γ and IL-17, in immune cells isolated from EAE mice [123]. In murine models of diet-induced atherosclerosis, a disease driven by inflammation and the generation of foam cells from macrophages, GPER-deficient mice exhibited increased aortic lesions and inflammation, whereas treatment of atherosclerotic wild-type mice with G-1 reduced macrophage infiltration and lesion extent [124]. In a contemporaneous report to this review, activation of GPER during pregnancy appears both necessary and sufficient to suppress IFN signaling, particularly in reproductive and fetal tissues, such that inactivation or inhibition of GPER in mice prevented fetal development and promoted fetal demise, specifically in the context of viral infection and maternal inflammation [125]. Thus, GPER could prove to be a valuable anti-inflammatory target in chronic inflammatory diseases, including multiple sclerosis and Crohn’s disease/ulcerative colitis [126], as well as acute inflammation during viral infections.

GPER also plays a critical role in metabolic regulation via modulation of inflammatory pathways as demonstrated by both murine models and epidemiological data [25,27]. GPER KO mice display higher levels of systemic pro-inflammatory cytokines, such as IL-1β, IL-6, monocyte chemoattractant protein-1 (MCP-1), serum amyloid A 3 (SAA3), and tumor necrosis factor-α (TNF-α), suggesting an anti-inflammatory role for the receptor [24,127]. In a mouse model of metabolic dysfunction and inflammation, treatment of ovariectomized (OVX) mice with G-1 resulted in decreased plasma levels of TNF-α, MCP-1, IL-6, leptin, and resistin, representing a reduced inflammatory state [25]. Furthermore, G-1 treatment resulted in decreased expression of inflammatory genes in metabolic tissues. *In vitro*, estrogen decreases the expression of pro-inflammatory genes in (pre)adipocytes, even following ERα knockdown, suggesting the presence of an ERα-independent mechanism, possibly involving GPER [128]. Collectively, these reports support an anti-inflammatory role for GPER in multiple settings, suggesting a possible role in other (patho)physiological settings including cancer, where both systemic and local (microenvironment) inflammation play important roles in carcinogenesis (Figure 1) [129].

## 7. GPER in Adipose/Adipocytes: Obesity and Influence on Cancer Development

In addition to fibroblasts and immune cells, the tumor microenvironment also contains adipocytes, which can be greatly enriched in fat-rich breast tissue [130,131]. Adipose tissue is no longer considered an inert energy storage component but is emerging as an active tissue involved in the secretion of various hormones and cytokines (adipokines) and the regulation of fuel availability, which together can play a key role in cancer development and progression (Figure 1) [131,132]. Adipose tissue and adipocytes are an integral part of both normal and tumor breast tissue along with immune cells, soluble factors, and the extracellular matrix. Evidence suggests extensive cross-talk between breast cancer cells and local adipocytes, as well as fat depots throughout the body, with obesity promoting breast cancer development in the post-menopausal woman [133]. Obesity leads to a chronic state of inflammation and persistent stress in white adipose tissue (WAT) that enhances carcinogenesis [132]. Furthermore, tumor growth and aggressiveness are enhanced by obesity due to the increased availability of fatty acids and other metabolites from adipocytes, aberrant adipokine signaling patterns, and associated immune dysfunction [131,134,135]. Mature adipocytes provide adipokines and lipids to cancer cells, whereas stromal and immune cells from adipose tissue infiltrate carcinomas to promote a pro-tumorigenic microenvironment [133].

GPER plays an important role in body weight regulation through fat deposition and accumulation. GPER-deficient mice exhibit increased overall body fat [24], whereas in diet-induced obese (DIO) and OVX mice, activation of GPER by its agonist G-1 results in reduced adiposity [25]. G-1 also promoted the expression of genes involved in mitochondrial biogenesis and fatty acid oxidation in WAT, suggesting a change in fuel utilization [25]. In 3T3-L1 cells, estrogen attenuates the adverse effects of inflammation on mitochondrial function by activating the protein kinase A pathway through GPER [136]. Furthermore, treatment of 3T3-L1 preadipocytes with estrogen or G-1 during differentiation inhibited lipid accumulation in adipocytes through the perturbation of mitotic clonal expansion, which was reversed by GPER knockdown [137].

Carcinogenesis is a highly complex multi-stage process in which GPER may exhibit multiple important roles. Based on the current understanding of the field, GPER could influence cancer development through both direct and/or indirect effects on WAT. GPER activation can exert differential effects on multiple cell types in breast tumors, promoting an anti-inflammatory phenotype and the oxidation of fatty acids [137]. The resulting smaller adipocytes, with reduced adipokine production, could possibly limit the availability of lipids and nutrients to breast tumors and inhibit their growth. Lastly, treatment with G-1 improves insulin sensitivity and lowers fasting plasma glucose and insulin levels in DIO and OVX mice [25]. An association between plasma insulin/IGF-1 concentrations and tumor development is widely acknowledged [138]. Thus, GPER activation may lower the plasma concentrations of insulin, thereby lowering the risk of cancer development. Therefore, GPER may regulate tumor progression by altering various functions in metabolic tissues.

## 8. Therapeutic Potential of Targeting GPER

Over the past several years, increasing evidence suggests that GPER could serve as a potential target for therapeutic intervention in several diseases. In the ERα-positive breast cancer setting, the cross-activation of GPER by the clinically widely used, ERα-targeted drug tamoxifen is hypothesized to contribute to the development of tamoxifen-resistant relapse tumors [17,18]. Combinatorial treatment of tamoxifen and the GPER inhibitor, G15, “re-sensitizes” tamoxifen-resistant tumors to tamoxifen [17]. To this end, the GPER-selective antagonists, G15 or G36 could potentially prove beneficial as part of a combinatorial therapy, with tamoxifen, for ERα-positive (relapse) breast cancer patients. Aside from GPER-inhibiting combinatorial therapies, an ERα antagonist that also inhibits GPER could prove beneficial for this class of breast cancer patient. To date, one study has identified the only known compound, termed MIBE, that exhibits such dual antagonist activity (i.e., antagonism of both ERα and GPER) [53]. Unfortunately, this compound shows poor binding affinity for ERα (IC_50_ > 10 μM), making it a poor candidate for clinical development. Nevertheless, MIBE could serve as a foundation for future ERα/GPER dual antagonists. Aside from pharmacological inhibition of GPER, inhibiting ERα in the absence of cross-activating GPER, could also be a viable pharmacological option for the treatment of ERα-positive breast cancers [64]. Such a compound could delay the development of ERα-positive relapse tumors. ERα-targeted antagonists that harbor such a molecular profile (i.e., high selectivity towards ERα with no selectivity towards GPER) have yet to be reported, but the identification of the ERα-selective/GPER non-selective agonist, AB-1, demonstrates that such molecular selectivity is achievable [56].

Whereas inhibition of GPER (or lack of GPER cross-activation) has possible therapeutic value for treating ERα-positive breast cancers, activation of the receptor could prove beneficial in the treatment of triple-negative breast cancers (TNBCs). G-1-mediated activation of GPER, in TNBC cell lines, results in cell cycle arrest and apoptosis, suggesting a protective role for GPER in TNBCs [139,140]. G-1 also inhibits tumor growth in xenograft models of TNBC, further supporting a case for GPER as a target in TNBC [23]. Although activation of GPER, in TNBC, appears beneficial [141,142], the field is still divided on whether GPER promotes or inhibits TNBC growth due to contradictory findings that show a pro-tumorigenic role for GPER in TNBC [143,144]. Nevertheless, given the lack of targeted therapies for TNBC, GPER-targeted therapies (agonists or antagonists) warrant further exploration.

GPER-targeted therapy has also garnered interest in the field of melanoma, with the demonstration that a combinatorial treatment of anti-PD-1 and G-1 increased the efficacy of the anti-PD-1 monotherapy in a murine model of melanoma, resulting in significantly smaller melanoma tumors and increased survival of the mice [22]. This discovery has led to the initiation of the first G-1 Phase I clinical trial (NCT04130516) by Linnaeus Therapeutics Inc. with the hope of advancing this drug to further clinical testing as a potential therapy for the treatment of melanoma and other cancers.

Therapeutic targeting of GPER may also be beneficial in metabolic dysfunction, such as obesity and diabetes. Selective activation of GPER by G-1, in murine models of obesity and metabolic dysfunction, exerts beneficial effects and mitigates obesity and diabetes. Mice exhibited reduced adiposity, inflammation, fasting glucose, insulin and cholesterol levels, with improved insulin sensitivity [25]. Despite G-1 mimicking a majority of the beneficial metabolic effects of E2, it lacks the classical feminizing effects of E2, as assessed by uterine imbibition. These results indicate that G-1 could be a drug of choice for metabolic dysfunction in males as well. Overall, GPER agonists exert multiple and differential effects in numerous tissues to regulate body weight and maintain metabolic homeostasis. These effects may have favorable outcomes in terms of tumor development and progression due to reduced substrate availability and blunted insulin signaling and inflammation. Thus, GPER-selective agonists may represent potential therapeutic candidate drugs for the treatment of obesity, diabetes, and certain cancers.

## 9. Conclusions

Estrogens play critical roles in both health and disease. Therapeutic regulation of its non-classical estrogen receptor GPER harbors potential clinical benefits in treating diseases in various tissue types, including adipose, skin, breast, and liver. However, the benefits of therapeutic activation or inhibition of GPER have been questioned in certain tissue types, specifically breast tissue, in the case of TNBCs, where some data support a proliferative effect of GPER activation. Nevertheless, data have shown therapeutic benefits of the GPER-selective agonist G-1 in multiple disease models, ranging from multiple sclerosis to atherosclerosis to melanoma and pancreatic cancer, to diabetes and obesity. With the initiation of the first Phase I clinical trial of G-1, the prospects for clinical targeting of GPER are promising and only time and additional research will tell if this approach will have a positive impact on human disease.
